# Investigation of the individual genetic evolution of SARS-CoV-2 in a small cluster during the rapid spread of the BF.5 lineage in Tokyo, Japan

**DOI:** 10.3389/fmicb.2023.1229234

**Published:** 2023-09-06

**Authors:** Bo Jin, Rieko Oyama, Yoko Tabe, Koji Tsuchiya, Tetsuya Hando, Mitsuru Wakita, Yan Yan, Mizue Saita, Satomi Takei, Yuki Horiuchi, Takashi Miida, Toshio Naito, Kazuhisa Takahashi, Hideoki Ogawa

**Affiliations:** ^1^Department of Clinical Laboratory, Peking University First Hospital, Beijing, China; ^2^Department of Research Support Utilizing Bioresource Bank, Juntendo University Graduate School of Medicine, Tokyo, Japan; ^3^Department of Clinical Laboratory Medicine, Juntendo University Graduate School of Medicine, Tokyo, Japan; ^4^Department of Clinical Laboratory, Juntendo University Hospital, Tokyo, Japan; ^5^Department of General Medicine, Juntendo University Graduate School of Medicine, Tokyo, Japan; ^6^Department of Respiratory Medicine, Juntendo University Graduate School of Medicine, Tokyo, Japan; ^7^Department of Dermatology, Juntendo University Graduate School of Medicine, Tokyo, Japan

**Keywords:** SARS-CoV-2, omicron variant, BF.5 lineage, gene mutation, cluster, whole genome sequencing, intra-host evolution, within-host diversity

## Abstract

There has been a decreasing trend in new severe acute respiratory syndrome coronavirus 2 (SARS-CoV-2) cases and fatalities worldwide. The virus has been evolving, indicating the potential emergence of new variants and uncertainties. These challenges necessitate continued efforts in disease control and mitigation strategies. We investigated a small cluster of SARS-CoV-2 Omicron variant infections containing a common set of genomic mutations, which provided a valuable model for investigating the transmission mechanism of genetic alterations. We conducted a study at a medical center in Japan during the Omicron surge (sub-lineage BA.5), sequencing the entire SARS-CoV-2 genomes from infected individuals and evaluating the phylogenetic tree and haplotype network among the variants. We compared the mutations present in each strain within the BA.5 strain, TKYnat2317, which was first identified in Tokyo, Japan. From June 29^th^ to July 4^th^ 2022, nine healthcare workers (HCWs) tested positive for SARS-CoV-2 by real-time PCR. During the same period, five patients also tested positive by real-time PCR. Whole genome sequencing revealed that the infected patients belonged to either the isolated BA.2 or BA.5 sub-lineage, while the healthcare worker infections were classified as BF.5. The phylogenetic tree and haplotype network clearly showed the specificity and similarity of the HCW cluster. We identified 12 common mutations in the cluster, including I110V in nonstructural protein 4 (nsp4), A1020S in the Spike protein, and H47Y in ORF7a, compared to the BA.5 reference. Additionally, one case had the extra nucleotide-deletion mutation I27* in ORF10, and low frequencies of genetic alterations were also found in certain instances. The results of genome sequencing showed that the nine HCWs shared a set of genetic mutations, indicating transmission within the cluster. Minor mutations observed in five HCW individuals suggested the emergence of new virus variants. Five amino acid substitutions occurred in nsp3, which could potentially affect virus replication or immune escape. Intra-host evolution also generated additional mutations. The cluster exhibited a mild disease course, with individuals in this case, recovering without requiring any medical treatments. Further investigation is needed to understand the relationship between the genetic evolution of the virus and the symptoms.

## Introduction

On November 24^th^ 2021, the World Health Organization (WHO) designated the Omicron variant of the SARS-CoV-2 as a variant of concern (VOC) due to its significant impact on global incidences (Gowrisankar et al., 2022; Quarleri et al., 2022; Vitiello et al., 2022). The Omicron variant carries specific mutations in the receptor binding domain (RBD), affecting its transmissibility and pathophysiology (He et al., 2021; Kim et al., 2021). Rapid structural changes and sub-lineages, including BA.1, BA.2, BA.3, BA.4, and BA.5, have become prevalent, enabling the virus to escape immunity from vaccinations or previous infections (Yao et al., 2022; Chatterjee et al., 2023). Since July 2022, the Omicron BA.5 subvariant has been dominant in Japan (National institute of infectious diseases, 2022, 99th Meeting of the COVID-19 Advisory Board of the Ministry of Health, Labour and Welfare^1^). Analysis of Omicron gene mutations revealed alterations in key ACE2 binding residues, resulting in reduced affinity (Wu et al., 2022) and increased immune-escape potential (Kumar et al., 2023). However, there is still limited understanding of the origin and the process of within-host genetic mutations (Miranda et al., 2022).

In this study, we observed the rapid emergence of the highly transmissible Omicron PANGO lineage (Rambaut et al., 2020), BF.5 (Ahmadi et al., 2022), in a small cluster transmission among healthcare workers (HCWs) within a span of 7 days. Numerous studies have identified distinct characteristics of intra-host genomic variation in the majority of SARS-CoV-2 infections. In MERS-CoV, a highly similar consensus sequence was observed (Borucki et al., 2016), while SARS-CoV-1 exhibited a higher mutation rate (Campbell et al., 2018). The understanding of mutations and transmission dynamics in SARS-CoV-2 is still evolving. These variations are commonly observed at low frequencies and are primarily influenced by purifying selection within the infected host. Additionally, they exhibit unique biochemical signatures (Graudenzi et al., 2021; Sapoval et al., 2021; Tonkin-Hill et al., 2021).

In this study, we aimed to investigate the rapid development of a specific SARS-CoV-2 cluster and the emergence of mutations within this group. We performed whole-genome sequencing analysis on the infected cases to gain insight into the variation of within-host diversity, mutational patterns, and selection pressures exerted on the viruses. Furthermore, we examined unique biochemical signatures associated with these variations by referring to previous studies and literature. Our findings provided valuable insight into the dynamics of mutations and transmission patterns in SARS-CoV-2.

## Materials and methods

### Description of hospital and baseline infection control measures

Juntendo University Hospital (JUH) has implemented strict infection control measures since the onset of the COVID-19 pandemic. The hospital mandates masking for all HCWs and patients in all facilities, including outpatient clinics and wards. All inpatients undergo real-time polymerase chain reaction (real-time PCR) testing from nasopharyngeal or saliva samples upon admission. HCWs, in addition to masks, are required to wear face shields or eye protection when interacting with patients, and N95 respirators are required when caring for patients with confirmed or suspected SARS-CoV-2.

### Collection of respiratory specimens and real-time PCR

Nasopharyngeal or saliva tests were conducted due to their high sensitivity and specificity (Yokota et al., 2020). The nasopharyngeal swabs were collected according to the standardized procedure^2^ (World Health Organization, 2006). The samples were subjected to RNA purification as described previously (Hosaka et al., 2023). The 2019 novel coronavirus detection kit (nCoV-DK, Shimadzu corporation, Kyoto, Japan) was used with the “2019-nCoV_N1” primer. Purified RNA samples were stored at −80°C for analysis.

### Next generation sequencing

The purified RNAs were reverse transcribed using the SuperScript VILO cDNA Synthesis Kit (Invitrogen, Carlsbad, CA, USA). The resulting cDNA was amplified with the Ion AmpliSeq SARS-CoV-2 Research Panel (Thermo Fisher Scientific, Waltham, MA, USA) on the Ion GeneStudio S5 System. This panel targets 237 amplicons in the SARS-CoV-2 genome and includes human expression control primers. Sequencing was performed on Ion GeneStudio S5 system using the Ion 530 chip (Thermo Fisher Scientific). Data analysis was conducted using Torrent Suite 5.14.0 and COVID19AnnotateSnpEff (v.1.3.0) plugin for variant annotation. SARS-CoV-2 variants with frequencies of single nucleotide polymorphisms (SNPs) higher than 18% or indel higher than 25% can be reproducibly detected with sequencing depth. All analyzed sequences had base accuracy exceeding 96% and base coverage exceeding 45-fold. The sequencing reads were subsequently submitted as FASTA files and deposited into the EpiCoV database of the Global Initiative on Sharing All Influenza Data (GISAID)^3^ with a reference (Wuhan-Hu-1, GenBank accession MN908947.3) (Shu and McCauley, 2017). SARS-CoV-2 lineages were assigned using the pangolin software (PANGO lineage v.3.1.15; O’Toole et al., 2021).

### Comparison of mutations

Nucleotide mutations and amino acid substitutions were obtained through variant call files from NGS data and the registered GISAID EpiCoV datasets. The changes were confirmed by visualizing the BAM files from the sequence fragment data using Integrated Genomics Viewer (IGV) v2.15.4 software (Robinson et al., 2011). The IGV is a widely used tool for exploring and interpreting genomic data, providing an interactive interface for examining sequence alignments, read coverage, and variant calling. The mutations were inspected on the IGV, accompanied by nucleotide and amino acid sequences from the reference Wuhan-Hu-1 sequence, to confirm codon usage. The Omicron BA.5 strain first observed in Tokyo on June 9^th^ was used as a reference for comparison (TKYnat2317).

### Phylogenetic tree and haplotype network analysis

A phylogenetic tree was constructed for the 14 cases by trimming poorly aligned regions in the 5′ and 3′ ends, as well as internal regions, based on the reference genome Wuhan-Hu-1. The fasta formatted sequences data were used to include positions ranging from the 55th to the 29,701st, and the internal trimming was performed on all sequences at the same positions used for the phylogenetic tree construction procedure. The trimmed sequences were then aligned using MAFFT (v7; Katoh et al., 2002) for tree construction. The maximum likelihood phylogenetic analysis was conducted using IQ-TREE v2.2.0, and the best-fit model was automatically selected with ultrafast bootstrap support values calculated from 1,000 replicates^4^ (Trifinopoulos et al., 2016; Kalyaanamoorthy et al., 2017). The resulting phylogenomic datasets were visualized using iTOL v6.7.3 (Letunic and Bork, 2021). Haplotype network data was generated with DnaSP v6.12.03, and the median-joining network was constructed by PopART v1.7 (Leigh and Bryant, 2015; Rozas et al., 2017). Reference sequences from GISAID, including Wuhan-Hu-1, the Omicron BA.1.17 strain (NICD-N21604-DX64219), and the BA.5, were used for phylogenic comparison.

### Isolation of low-frequency mutations

The NGS data analysis was conducted using Torrent Suite. The variant caller protocol parameters included a minimum allele frequency higher than 5% for indels, SNPs, and multiple nucleotide polymorphisms (MNPs). Additionally, a minimum alternative allele frequency and a minimum indel alternative allele frequency higher than 2.5% were applied. These values were determined based on the recommendations provided by the manufacturer. Variants were identified using the Wuhan-Hu-1 reference genome and annotated with the SARS-CoV-2 AnnotateSnpEff plugin. The alignments were visually inspected using IGV to detect false positives. Nucleotide and amino acid changes within the cluster-related viruses were summarized.

## Results

**Figure 1:**
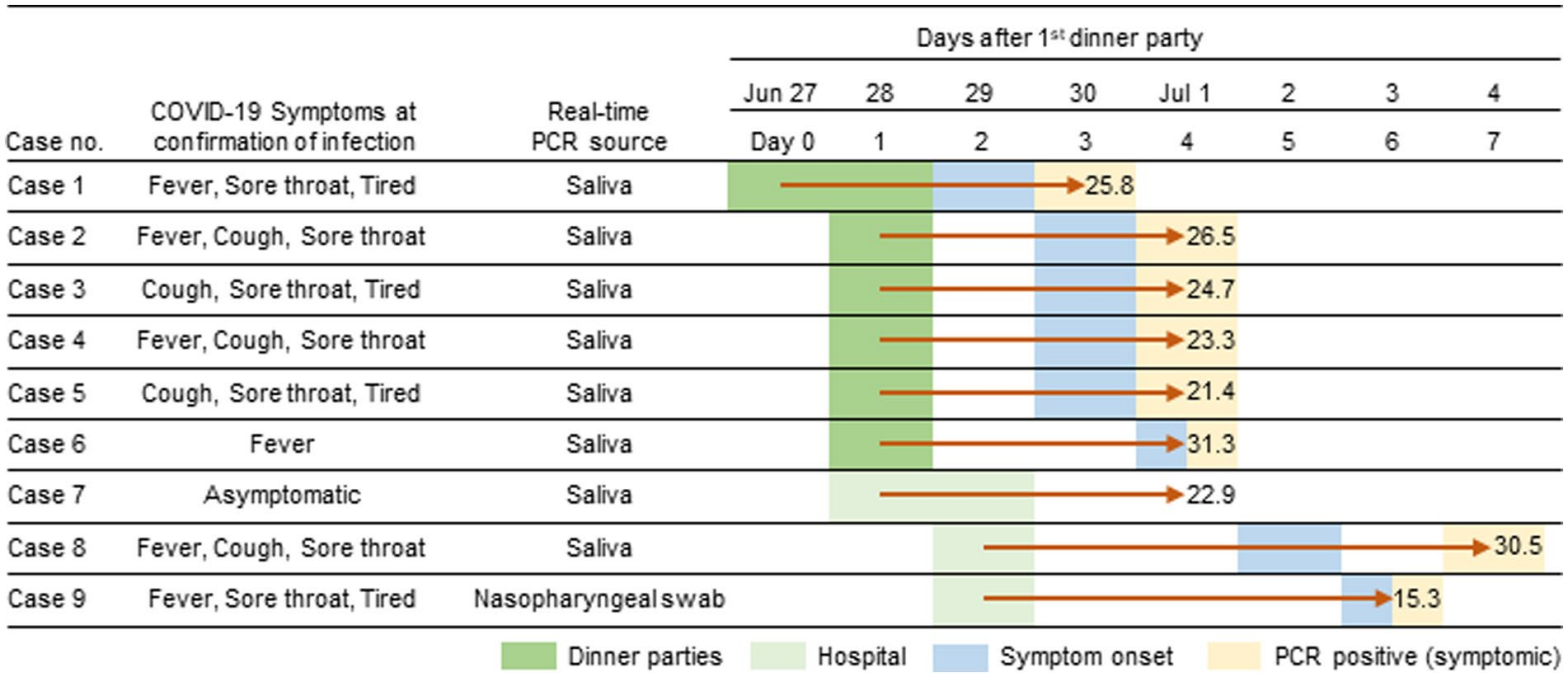
Timeline of the Omicron BF.5 outbreak among healthcare workers (HCWs) at the JUH. On June 27^th^ and 28^th^, six HCWs attended a dinner gathering party. Three HCWs joined the department on June 28^th^ and 29^th^. The first symptom onset was reported on June 29^th^ and confirmed positive by real-time PCR the following day. All HCWs tested positive within a week after the initial infection. The duration between the gathering and diagnosis in indicated by red arrows. CT values of the test are provided.

From June 30^th^ through July 4^th^ 2022, 9 HCWs of JUH, aged between 25 and 33, were diagnosed with SARS-CoV-2 infections (highlighted in light orange, Figure 1). Among these HCWs, six had participated in a meal gathering on June 27^th^ and 28^th^ (highlighted in green, Figure 1), while the remaining three HCWs did not attend the gathering but worked at the same ward (highlighted in light green, Figure 1). Case 1, who attended the meal gathering on June 27^th^ and 28^th^, developed COVID-19 symptoms first on June 29^th^ and tested positive on June 30^th^. Following case 1, five other HCWs who attend the meal gathering on June 28^th^ (referred to as case 2–6) also developed symptoms on June 30^th^ or July 1^st^ and tested positive for COVID-19 on July 1^st^. In addition, three HCWs who did not attend the gathering but worked in the same ward (cases 7–9) also tested positive for COVID-19 during July 1^st^ and 4^th^. In each HCW except for case 7, symptoms appeared within 7 days, and none required hospitalization. Six HCWs were fully vaccinated (two doses, in 2022 Tokyo), two were partially vaccinated (one dose), and one was unvaccinated (Table 1).

**Table 1:** Characteristics of infected healthcare workers (HCWs) in the nosocomial cluster, June 29 through July 4, 2022.

Case no.	Pango lineage	Gender	Age	Vaccination
Case 1	BF.5	Female	30	1
Case 2	BF.5	Female	25	2
Case 3	BF.5	Female	27	2
Case 4	BF.5	Male	27	1
Case 5	BF.5	Male	29	2
Case 6	BF.5	Male	33	2
Case 7	BF.5	Male	27	2
Case 8	BF.5	Female	28	2
Case 9	BF.5	Female	33	0

The number in the “Vaccination” column represents the count of vaccine doses received. This table reflects the maximum number of vaccine doses (two doses) available in Japan during mid-2022.

**Figure 2:**
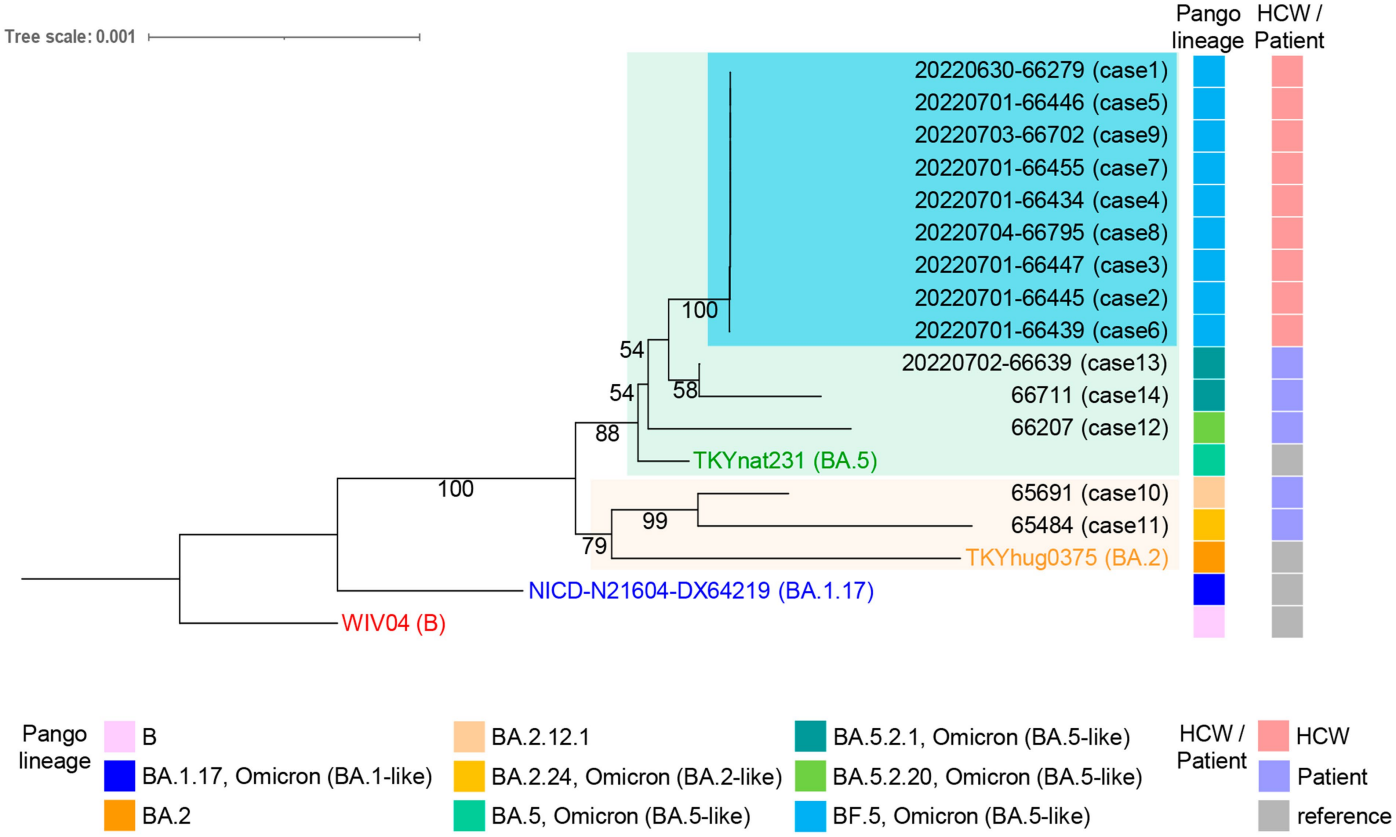
Whole-genome phylogenetic analysis of SARS-CoV-2 genomes from individuals with onset between June 16^th^ and July 3^rd^ at the JUH. The HCWs (cases 1–9) cluster, highlighted in light blue, shows a distinction from the five patients (cases 10–14) in the tree. The maximum log-likelihood of this tree is −38077.05 under the best-fit model HKY + F + I selected in IQ-TREE v2.2.0 and visualized using iTOL v6.7.3. Each sample is labeled with the virus name and the case number. The ultrafast bootstrap support values are shown in the constructed tree. Pango lineage and HCW or patient classification are shown on the right. The virus name of Wuhan-Hu-1 (WIV04, GenBank accession number MN908947.3, EPI_ISL_402124), the Omicron BA.1.17 strain (NICD-N21604-DX64219, EPI_ISL_6647959), the BA.2 strain (THYhug0375, EPI_ISL_9890267), and the BA.5 strain (TKYnat231, EPI_ISL_13843385) were indicated and used as references.

In the same period as the HCW cluster, five patients tested positive between June 22^nd^ and July 3^rd^ at JUH (Supplementary Table 1). The five patients had no record of contact with the HCWs. Their outpatient visit date and hospitalization periods were indicated in Supplementary Table 1. We included the data from these patients for comparison with the HCW cluster in this study to explore the transmission dynamics and investigate potential hospital-acquired infections. The RNA samples were also prepared. Two patients had been fully vaccinated (two doses, Supplementary Table 1, cases 10, 12); two were partially vaccinated (one dose, cases 13, 14); one was without an available vaccination record (case 11). The SARS-CoV-2 RNA samples from the 14 infection cases were sequenced using NGS. The sequences from the HCWs all belonged to the BF.5 PANGO lineage. In contrast, the sequences from patients who experienced symptom onset on Jun 16^tth^ and 22^nd^ were found to be infected with BA.2.12.1 and BA.2.24 variants, respectively (case 10,11, Figure 2). The patient who experienced symptom onset on June 26^th^ was infected with the BA.5.2.20 variant (case 12), while the patients who experienced onsets on June 30^th^ were infected with the BA.5.2.1 variant (cases 13 and 14).

Mutations in the 9 HCWs sequences shared 12 common genomic changes and four amino acid substitutions compared to the BA.5 sequence (Table 2). All cases have genomic nucleotide changes with no amino acid change at position V2238 in ORF1ab (nonstructural protein 3, nsp3), I2873V in ORF1ab (nsp4), no amino acid change at position L3234 in ORF1ab (nsp4), no amino acid change at position L556 in ORF1ab (nsp13), no amino acid change at position L642 in ORF1ab (nsp14), A1020S in Spike protein, no amino acid change at position T172 in Membrane protein, H47Y in ORF7a, no amino acid change at position G19 and Q43 in Nucleocapsid protein, respectively. Five genomic mutations occurred in ORF1ab, and three amino acid substitutions were isolated. Truncation variant I27fs* in ORF10 was also observed in the case 6 HCW who had gathered for the meal. To confirm the accuracy of these mutations, we compared them with the nucleotide and amino acid sequences of the Wuhan-Hu-1 reference, focusing on codon usage using the BAM files and IGV software. The IGV analysis revealed that the mutations in the HCWs sequences demonstrated precise codon correctness (A-L, N for case 1, represented in Supplementary Figure 1). Furthermore, in case 6, we observed a specific decrease in the nucleotide count at position 2,9636 in the genome compared to the counts in other positions nearby, while the counts in other cases remained consistent (Supplementary Figure 1, M for case 6 compared with L for case 1), indicating one-nucleotide deletion. In contrast, mutations from the five patients varied from each other (Supplementary Table 2).

**Table 2:** Nucleotide and amino acid changes in HCWs compared to the BA.5 reference sequence.^a^

BA.5			Mutation type: Type 1			Mutation type: Type 2		
			Case 1, 2, 3, 4, 5, 7, 8, 9			Case 6		
Genome change	Amino acid change	Gene	Genome change	Amino acid change	Gene	Genome change	Amino acid change	Gene
			C44T		5′UTR	C44T		5′UTR
del509_523	G82_V86del	ORF1ab (nsp1)						
G2202T	W646L	ORF1ab (nsp2)						
G2747A	No change	ORF1ab (nsp3)										T6979G	No change	ORF1ab (nsp3)	T6979G	No change	ORF1ab (nsp3)				A8882G	I2873V	ORF1ab (nsp4)	A8882G	I2873V	ORF1ab (nsp4)				C9967T	No change	ORF1ab (nsp4)	C9967T	No change	ORF1ab (nsp4)				C16954T	No change	ORF1ab (nsp13)	C16954T	No change	ORF1ab (nsp13)				C19524T	No change	ORF1ab (nsp14)	C19524T	No change	ORF1ab (nsp14)				G24620T	A1020S	S	G24620T	A1020S	S
C25466T	No change	ORF3a						
A26295T	No change	E										A27038G	No change	M	A27038G	No change	M				C27532T	H47Y	ORF7a	C27532T	H47Y	ORF7a				A28330G	No change	N	A28330G	No change	N				A28402G	No change	N	A28402G	No change	N
C28744T	No change	N													del29636_29636	I27fs*	ORF10				del29734_29759		3’UTR	del29734_29759		3’UTR

a The nucleotide changes were compared with the Omicron BA.5 strain first reported in Tokyo, Japan (EPI_ISL_13843385). The Omicron BA.5 strain was used as a standard of comparison to the Wuhan-Hu-1 (GenBank accession number MN908947.3, EPI_ISL_402124).

**Figure 3:**
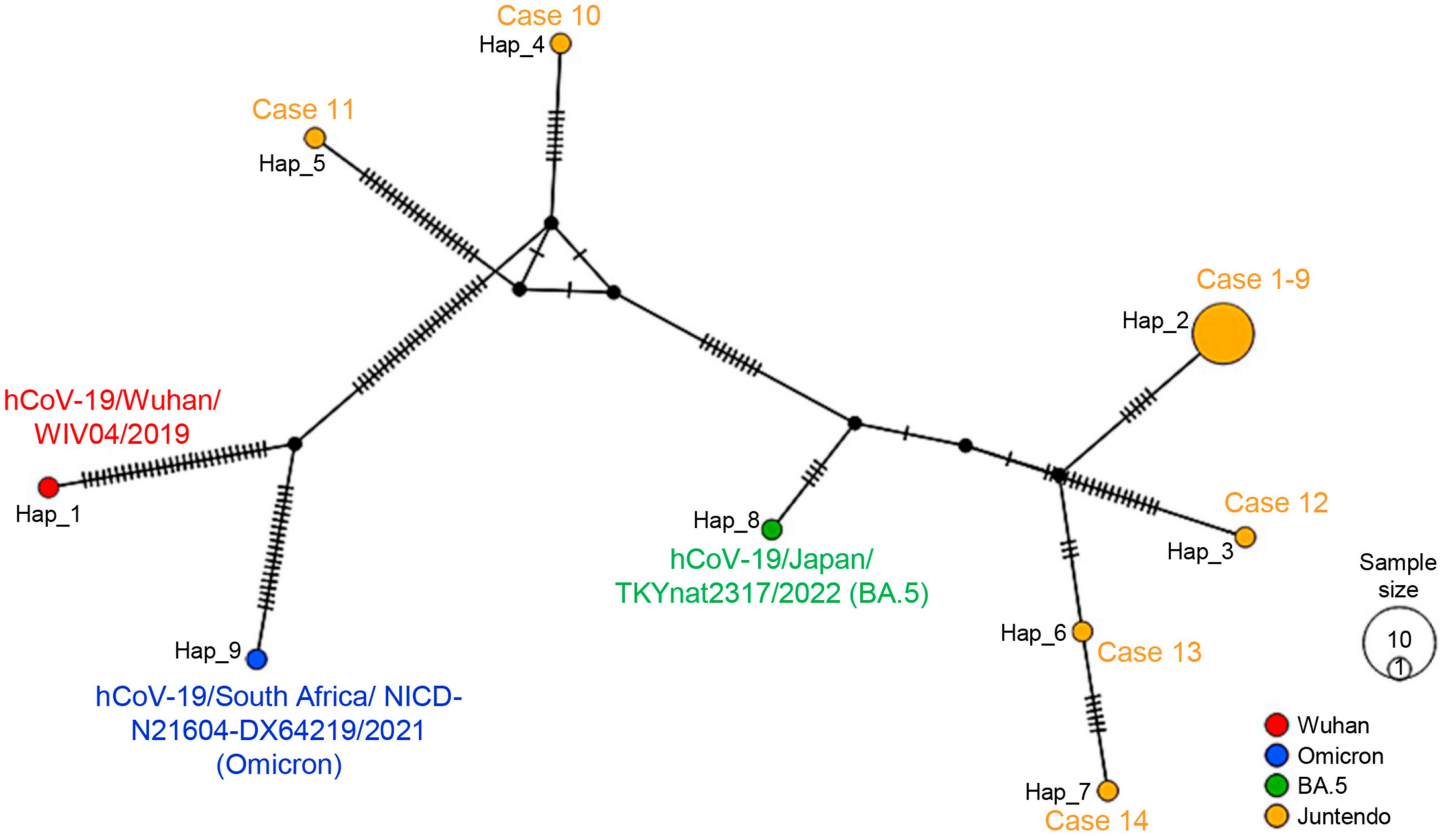
The haplotype network for all Omicron SARS-CoV-2 sequences from individuals with onset between June 16^th^ and July 4^th^ at JUH is shown. The size of each node is proportional to the number of samples that belong to that haplotype. The color represents the PANGO lineage of references or the case samples for each haplotype. The number of lines on the branch between each node corresponds to the number of mutations between them. Each haplotype was indicated by case number or reference. Data were analyzed DnaSP v6.12.03 and drawn by PopART v1.7. The following reference viruses were used: the Wuhan-Hu-1 (GenBank accession number MN908947.3, EPI_ISL_402124), the Omicron BA.1.17 strain (EPI_ISL_6647959), and the BA.5 strain (EPI_ISL_13843385).

The phylogenetic tree of the 14 samples was analyzed using the Wuhan-Hu-1, the Omicron BA.1.17, the BA.2, and the BA.5 sequences as references. The maximum log-likelihood of this tree was −38077.05 under the best-fit model HKY + F + I. The Blanching diagram shows the relationships among the cases: HCWs cases 1–9 are located in the branch block of the BA.5 reference. The five patients, who were not in direct contact with the HCWs, exhibited differences in their genetic clustering. Specifically, cases 12, 13, and 14 were found in the same branch block as the HCW cluster within the BA.5 reference, while cases 10 and 11 showed a separate branch block within the BA.2 reference (Figure 2). The high bootstrap values observed in the upper branches, such as a value of 100, strongly indicate a distinct separation between HCW cluster and the patients (cases 12–14) within the BA.5 branch block. The HCW cluster is a sister group in the BA.5 sub-lineage, showing a high degree of genetic similarity with low bootstrap values (Figure 2). The alignment sequences using the phylogenic tree were compared with the nucleotide and amino acid reference sequence Wuhan-Hu-1, encompassing the entire genome sequence to ensure the preservation of codon usage (Supplementary Dataset 1). This process was also summarized in Supplementary Dataset 2, which includes nine out of 12 common mutations in the HCW cluster retained in the alignments (indicating hatch boxes in Supplementary Dataset 2). The phylogenetic analysis extended to the spike protein sequences, translated from the trimmed genomic sequences, further highlighting the distinctiveness of the HCW cluster in comparison to patients (Supplementary Figure 2, Supplementary Data 3). In the haplotype network analysis, the HCW cluster was aggregated into a single haplotype, indicating that the individuals in this cluster were closely related (Figure 3). Cases 12–14 had unique haplotypes derived from the BA.5 reference, which differed from those of the HCWs cases 1–9. Furthermore, patient cases 10 and 11 showed variations from the Wuhan-1 and Omicron references (Figure 3). This comprehensive analysis corroborates the robustness of our approach and enhances the validity of our findings, both at the genomic and protein levels.

Low-frequency genomic variants in the HCWs case 1–9 cluster compared to the BA.5 reference sequence are shown in Table 3. A total of seven minor variants were identified, mostly with changes to T (Table 3, C4947T, G7406T, C13536T, C21331T) in cases 4, 5, 6, and 8. Among the HCWs who attended a meal gathering, minor variants were identified in cases 4, 5, and 6, while not in cases 1, 2, and 3. In case 8, the minor variants were isolated at four positions. Y1423fs*88 in ORF1ab (nsp3), V2381F in ORF1ab (nsp3), Y2391fs*91 in ORF1ab (nsp3), and the no amino acid change at position Y4424 in ORF1ab (nsp12). In case 6, there were three minor variants: S1161fs*16 in ORF1ab (nsp3), no amino acid change at position A3045 in ORF1ab (nsp4), and the no amino acid change at position Y4424 in ORF1ab (nsp12). In case 5, a minor variant S1561F was observed in ORF1ab (nsp3). In cases 7 and 9, who did not attend any meal parties, no minor variants were observed. The no amino acid change at position Y4424 (C13536T in the genome) variant is shared in cases 4, 6, and 8.

**Table 3:** Minor variants in the HCW cluster-related viruses.^a^

Case No.	Mutation type	Position (Wuhan-Hu-1 numbering)	Ref	Alt	Read depth	Frequency	Variant type	Gene		Amino acid modification
Case 1	Type 1	N/A								
Case 2	Type 1	N/A								
Case 3	Type 1	N/A								
Case 4	Type 1	13536	C	T	36525	0.31	synonymous_variant	ORF1ab	(nsp12)	No change
Case 5	Type 1	4947	C	T	21331	0.23	missense_variant	ORF1ab	(nsp3)	S1561F
Case 6	Type 2	3745	TTC	T	968	0.16	frameshift_variant	ORF1ab	(nsp3)	S1161fs*16	9400	A	G	558	0.19	synonymous_variant	ORF1ab	(nsp4)	No change	13536	C	T	12010	0.49	synonymous_variant	ORF1ab	(nsp12)	No change
Case 7	Type 1	N/A								
Case 8	Type 1	4528	A	AT	2916	0.23	frameshift_variant	ORF1ab	(nsp3)	Y1423fs*88	7406	G	T	1247	0.13	missense_variant	ORF1ab	(nsp3)	V2381F	7435	TTATTA	T	5130	0.11	frameshift_variant	ORF1ab	(nsp3)	Y2391fs*91	13536	C	T	62248	0.52	synonymous_variant	ORF1ab	(nsp12)	No change
Case 9	Type 1	N/A								

a SARS-CoV-2 strain Wuhan-Hu-1 (GenBank accession number MN908947.3, EPI_ISL_402124) was used as the reference genome for mapping reads.

## Discussion

In this study, we observed transmission with common mutations of the SARS-CoV-2 Omicron BF.5 PANGO lineage among nine individuals over 7 days at JUH. This cluster shared 12 genomic alterations, including three amino acid substitutions. The transmission period coincided with a previous study conducted in Japan, where the Omicron BA.1 sub-lineage virus was infectious 0–2 days after the symptoms disappeared (Takahashi et al., 2022).

The HCW cluster was distinguishable from the patients infected with the Omicron during the same period based on the virus genome sequences, such as variants, PANGO lineage, and relationships in the phylogenetic tree and haplotype network. In Japan, the dominant strain of SARS-CoV-2 shifted from the BA.2 sub-lineage to the BA.5 sub-lineage from June to August 2022, as indicated by records in the GISAID EpiCoV database. Between June 28^th^ and July 10^th^, the BF.5 lineage accounted for 955 of the total 10,868 cases in Tokyo. The BF.5 lineage was ranked among the top three most common in Japan, along with BA.5.2 and BA.5.2.1, in July 2022, indicating its rapid spread. Between October 2022 and February 2023, BF.5 was the most common lineage according to GISAID. This cluster had three substitution mutations: I2873V in ORF1ab (I110V in nsp4), A1020S in the S protein, and H47Y in ORF7a (Table 2). The A1020S mutation in the S protein is commonly observed in the BF.5 lineage. Variants I2893V in ORF1ab (I110V in nsp4) and H47Y in ORF7a are characteristic mutations in the BF.5.1 lineage (National Library of Medicine, 2023; SARS-CoV-2 Variants Overview, NCBI)^5^. These suggest that the viral genomes in the HCW cluster were developing from the BF.5 lineage to the BF.5.1 lineage.

The clinical samples have certain limitations due to the trimming performed to address poor reading depth. However, in our study, we carefully aligned the trimmed sequences with the nucleotide and amino acid reference sequence Wuhan-Hu-1 to ensure the preservation of codon usage. This allowed us to confirm the accuracy and reliability of the mutations used in our phylogenetic analysis. Nine of twelve common mutations in the HCW cluster were retained in the trimmed sequences, reaffirming the specificity of the HCW cluster. Our approach enabled us to conduct a robust phylogenetic analysis despite the trimming, ensuring that crucial genomic information was not compromised. Despite these limitations, our phylogenetic tree clearly demonstrates a strong correlation with the PANGO lineage and its position within the branch block (Figure 2). The clustering and grouping of the HCW cluster in the phylogenetic tree, supported by higher bootstrap values, emphasize the distinct separation of the HCW cluster from other strains or patients within the BA.5 branch block. Despite the potential convenience of utilizing VIRULIGN (Libin et al., 2018) for aligning the translated protein sequences, our strategy involved applying the mapping-guided trimming approach practically (Supplementary Figure 2, Supplementary Data 3). This approach established a dependable basis for conducting our phylogenetic analysis and deriving meaningful insights from the obtained data. Furthermore, the haplotype network analysis supports the evolutionary dynamics of the virus among different lineages (Figure 3). Collectively, these analyses provide valuable insights into the relatedness and genetic diversity among the samples.

We conducted a comparative analysis of the HCW cluster and the patients who detected viruses during the same period to explore the transmission dynamics within the hospital setting. The phylogenetic and haplotype network analysis did not reveal direct connections between the HCW cluster and the patients. However, this comparison provided insights into potential links, shared genetic variations, and broader context of transmission within the hospital environment. Despite the lack of direct linkage, this comprehensive analysis supports the conclusion that the HCWs in the cluster were not directly linked to the patients in the hospital. This finding further enhances our understanding of the transmission landscape and the dynamics of viral spread within the hospital.

Additionally, it is worth noting that given the limited number of genetic mutations within this HCW cluster, the practicality and applicability of a BEAST analysis (Bouckaert et al., 2019) in this specific investigation may be restricted. However, for future studies involving more complex clusters, a BEAST analysis could potentially offer additional insights into phylogenetic uncertainty and evolutionary dynamics, and it may be necessary to further elucidate the evolutionary dynamics and transmission patterns.

During this transmission among the HCW cluster, a nucleotide deletion was observed in case 6, resulting in a frameshift variant and truncation at the 27th amino acid residue in ORF10 (Table 2). ORF10 is a unique protein consisting of 38 amino acid residues with no homologous proteins in coronaviruses (Wu et al., 2020). A proteomics study reported that ORF10 interacts with an E3 ubiquitin ligase complex responsible for degrading proteins through ubiquitination (Gordon et al., 2020). ORF10 suppresses the IFN-I signaling pathway by interacting with the mitochondrial antiviral signaling protein (MAVS), degraded via the ORF10-induced autophagy pathway. In addition, ORF10 interacts with Nip3-like protein X (NIX) and LC3B and induces mitophagy (Li et al., 2022). The I27fs* variant of ORF10 could potentially impact functions associated with proteosome-mediated proliferation and the immune system’s response. The CT value of 31.3 indicated a low-level proliferation within the host, suggesting a potential decrease in the function of ORF10.

This cluster showed nine minor variants. In ORF1ab, corresponding to nsp3, five amino acid substitution variants were identified, which were S11161fs*16 (case 6), Y1423fs*88 (case 8), S1561F (case 5), V2381F (case 8), and Y2391fs*91 (case 8). The ORF1ab or ORF1a proproteins are cleaved co- and post-translationally by two viral proteases, a papain-like protease (PLPro) residing in nsp3 and the 3C-like protease (Ziebuhr et al., 2000). Nsp3 protein, composed of 1,944 amino acid residues, is the largest protein with multiple membrane-spanning domains. It plays a role in replication and transcription process, forming a functional complex (Van Hemert et al., 2008; Lei et al., 2018). Nsp3 can function as a scaffold protein, facilitating interactions with itself as well as binding to other viral non-structural proteins or host proteins (von Brunn et al., 2007; Imbert et al., 2008). Case 5 showed minor mutations S1561F in ORF1ab. The residue S1561 is located within the region where the C-terminal SARS-Unique Domain (SUD) and PLPro domain contract or coexist (Neuman et al., 2008). The characteristic of the feature was not observed in this case. Further research and analysis would be necessary to investigate the potential effects of such substitutions. Case 6 shows a S1161fs*16 frameshift mutation in macrodomain I, X-domain of viral nsp3 and related macrodomains in ORF1ab proprotein. The X-domain is conserved in all coronaviruses for RNA replication (D'Amours et al., 1999). The frameshift deletion indicates the loss of functions associated with the replication-transcription complex and the coding region downstream of it, leading to impaired replication. Case 8 also exhibited the following mutations: Y1423fs*88, V2381F, and Y2391fs*91. The residue Y1423 is located within SARS-unique domain of single-stranded poly (A) binding domain (SUD-M; Snijder et al., 2003; Lei et al., 2018). The residues V2381 and Y2391 are located in the transmembrane and Y domains, respectively. The SARS-unique domain (SUD) and Y domain, which comprise the highly-conserved C-terminal region of nsp3, play a crucial role in interactions with potential ligands and other nsps (Li et al., 2023). These mutations suggest potential alterations in protein function. Furthermore, the frameshift deletion implies the loss of the functions and the coding region downstream of it. The CT value of 30.5 indicated a low-level proliferation within the host, suggesting a reduced viral replication potential. Additional studies are needed to gain a better understanding of the implications.

In this cluster, minor mutations mainly involved C/G > T substitutions in the genome (Table 3). In addition, specific nucleotide alterations were observed in this cluster compared to the BA.5 references, including A > G at four genomic positions and C > T at six genomic positions (Table 2). Consistent with the literatures (Graudenzi et al., 2021; Sapoval et al., 2021; Tonkin-Hill et al., 2021), there was a bias toward T nucleotide mutations in the host diversities. Furthermore, three cases exhibited a synonymous mutation C13536T (no amino acid change at Y4424) in nsp12 (Table 3). Cases 4 and 8 are closely related in the phylogenetic tree, while case 6 is more distantly related (Figure 2). It remains unclear whether these mutations were influenced by neighboring nucleotide sequences or occurred by chance. Overall, the variants in this cluster suggested less events during transmission.

Based on these observations, although T mutations are frequently observed, it is insufficient to predict whether these mutations will exacerbate the onset of SARS-CoV-2. In this cluster of healthy individuals, infection was rapid and several genetic mutations occurred throughout this cluster transmission, but the symptoms were not severe. Between October 2022 and February 2023, BF.5 reached the highest ranking according to GISAID. After March 2023, the XBB.1.5 variants became the most common despite a falling infection rate in Japan and worldwide. However, the virus has not yet disappeared, and further genetic changes need to be monitored since infection fluctuates seasonally and genetic evolution is expected to continue.

## Limitations

There are some limitations worth addressing. First, this is a single cluster study during the Omicron BA.5 PANGO sub-lineage surge in Tokyo with a limited number of samples. This study does not attempt to fully elucidate the mechanism of the Omicron variant’s evolution. Second, the small sample size did not allow us to identify a significant association between vaccination and mutation rate.

## Conclusion

A small cluster of individuals infected with an Omicron variant BF.5 lineage was identified among the healthy healthcare workers (HCWs) of JUH during the rapid spread in Tokyo in 2022. The cluster, characterized by shared mutations, spread within 1 week, and did not require medical treatment for recovery. Due to the limited spread and clear chain of transmission, extensive epidemiological surveys were not necessary. The presence of a unique variant in ORF10 and minor variants in ORF1ab within individuals suggests ongoing genomic evolution as the virus disseminated within the cluster. Monitoring these mutations is essential for effective response to future outbreaks.

## Data availability statement

The datasets presented in this study can be found in online repositories. The names of the repository/repositories and accession number (s) can be found at: https://www.ddbj.nig.ac.jp/, LC772113–LC772126.^6^

## Ethics statement

The studies involving humans were approved by Institutional Review Board (IRB) at Juntendo University Hospital, Japan (IRB #20-036). The studies were conducted in accordance with the local legislation and institutional requirements. The human samples used in this study were acquired from primarily isolated as part of your previous study for which ethical approval was obtained. Written informed consent for participation was not required from the participants or the participants’ legal guardians/next of kin in accordance with the national legislation and institutional requirements. Written informed consent was obtained from the individual (s) for the publication of any potentially identifiable images or data included in this article.

## Author contributions

YT had full access to all the data in the study and takes responsibility for the integrity of the data and the accuracy of the data analysis. BJ, RO, YT, KTs, TH, MW, YY, MS, ST, YH, TM, TN, KTa, and HO concept and design and critical revision of the manuscript for important intellectual content. BJ, RO, KTs, TH, YY, ST, YH, and YT acquisition, analysis, or interpretation of data. BJ, RO, and YT drafting of the manuscript. BJ and RO statistical analysis. MW, MS, TM, TN, KTa, and HO administrative, technical, or material support. TM, TN, KTa, and HO supervision. All authors contributed to the article and approved the submitted version.

## Funding

This research was supported by the Japan China Sasakawa Medical Fellowship (to BJ), Japan Agency for Medical Research and Development (JP20fk0108472 to TN) and by Japan Society for the Promotion of Science Grants-in Aid for Scientific Research (22K08608 to MS and 22K15675 to ST).

## Conflict of interest

The authors declare that the research was conducted in the absence of any commercial or financial relationships that could be construed as a potential conflict of interest.

## Publisher’s note

All claims expressed in this article are solely those of the authors and do not necessarily represent those of their affiliated organizations, or those of the publisher, the editors and the reviewers. Any product that may be evaluated in this article, or claim that may be made by its manufacturer, is not guaranteed or endorsed by the publisher.

## Footnotes

^1^ https://www.niid.go.jp/niid/en/2019-ncov-e/11525-covid19-ab99th-en.html

^2^ https://apps.who.int/iris/handle/10665/69392

^3^ https://gisaid.org/

^4^ http://www.iqtree.org/

^5^ https://www.ncbi.nlm.nih.gov/activ

^6^ http://getentry.ddbj.nig.ac.jp/
