# Supplementary material for: Investigation of the individual genetic evolution of SARS-CoV-2 in a small cluster during the rapid spread of the BF.5 lineage in Tokyo, Japan
Source: Front Microbiol. 2023 Sep 6;14:1229234. doi: 10.3389/fmicb.2023.1229234 (PMC10516552; doi:10.3389/fmicb.2023.1229234)
Supplement: Supplementary file 4 [file Presentation_1.PPTX]

## Slide 1
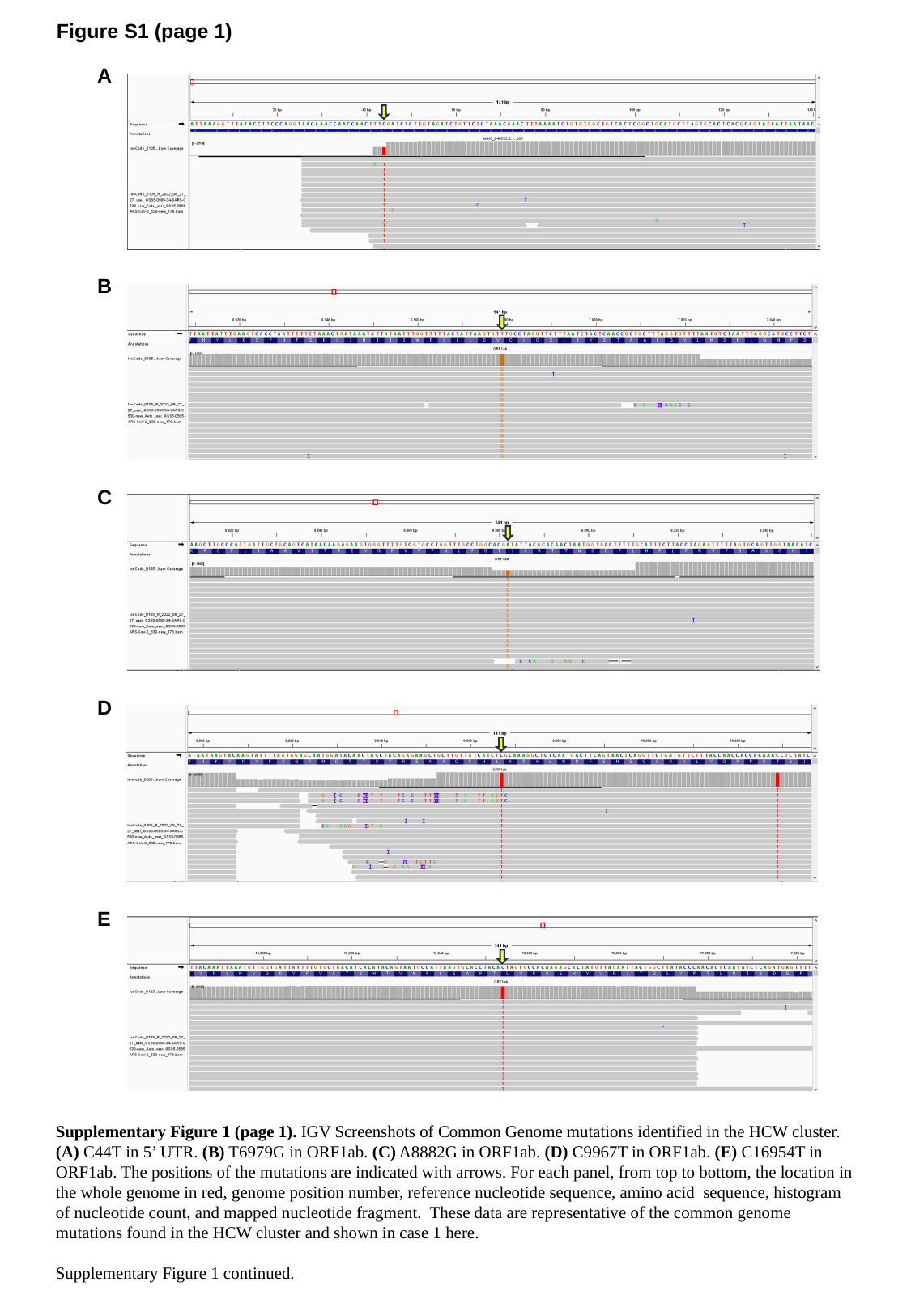

Figure S1 (page 1)
A
B
C
D
E
Supplementary Figure 1 (page 1). IGV Screenshots of Common Genome mutations identified in the HCW cluster.
(A) C44T in 5’ UTR. (B) T6979G in ORF1ab. (C) A8882G in ORF1ab. (D) C9967T in ORF1ab. (E) C16954T in ORF1ab. The positions of the mutations are indicated with arrows. For each panel, from top to bottom, the location in the whole genome in red, genome position number, reference nucleotide sequence, amino acid sequence, histogram of nucleotide count, and mapped nucleotide fragment. These data are representative of the common genome mutations found in the HCW cluster and shown in case 1 here.
Supplementary Figure 1 continued.

## Slide 2
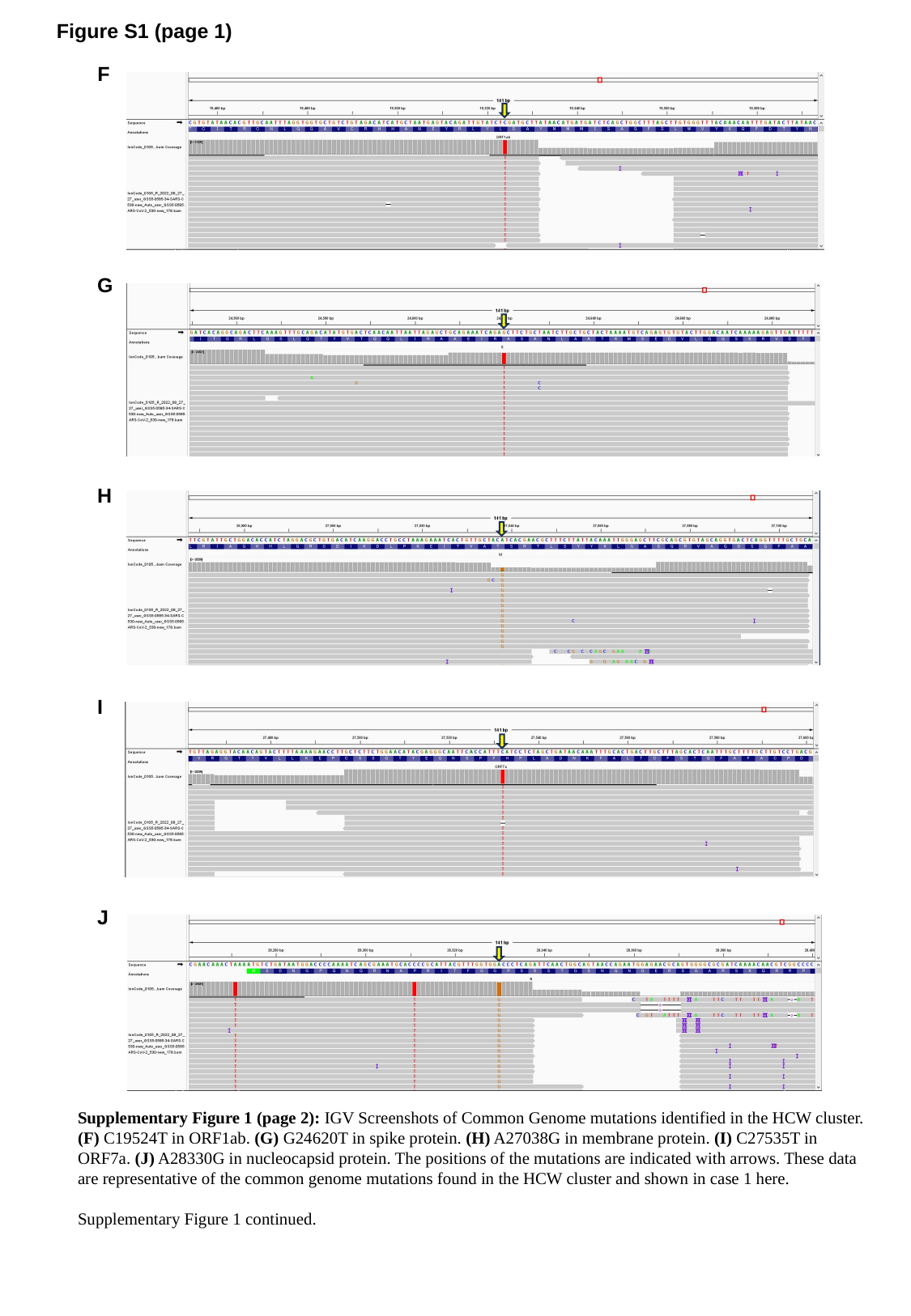

Figure S1 (page 1)
F
G
H
I
J
Supplementary Figure 1 (page 2): IGV Screenshots of Common Genome mutations identified in the HCW cluster.
(F) C19524T in ORF1ab. (G) G24620T in spike protein. (H) A27038G in membrane protein. (I) C27535T in ORF7a. (J) A28330G in nucleocapsid protein. The positions of the mutations are indicated with arrows. These data are representative of the common genome mutations found in the HCW cluster and shown in case 1 here.
Supplementary Figure 1 continued.

## Slide 3
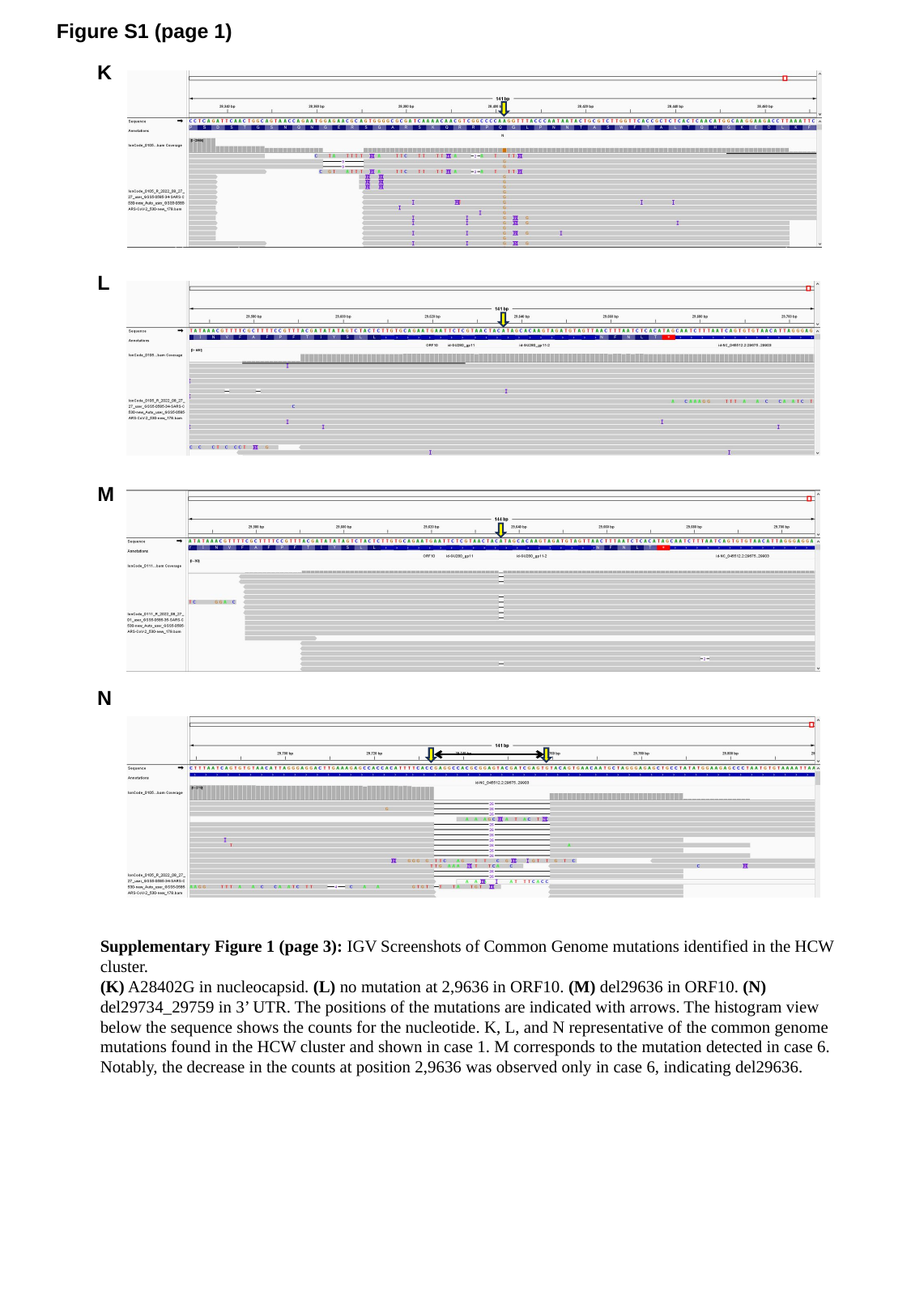

Figure S1 (page 1)
K
L
M
N
Supplementary Figure 1 (page 3): IGV Screenshots of Common Genome mutations identified in the HCW cluster.
(K) A28402G in nucleocapsid. (L) no mutation at 2,9636 in ORF10. (M) del29636 in ORF10. (N) del29734_29759 in 3’ UTR. The positions of the mutations are indicated with arrows. The histogram view below the sequence shows the counts for the nucleotide. K, L, and N representative of the common genome mutations found in the HCW cluster and shown in case 1. M corresponds to the mutation detected in case 6. Notably, the decrease in the counts at position 2,9636 was observed only in case 6, indicating del29636.

## Slide 4
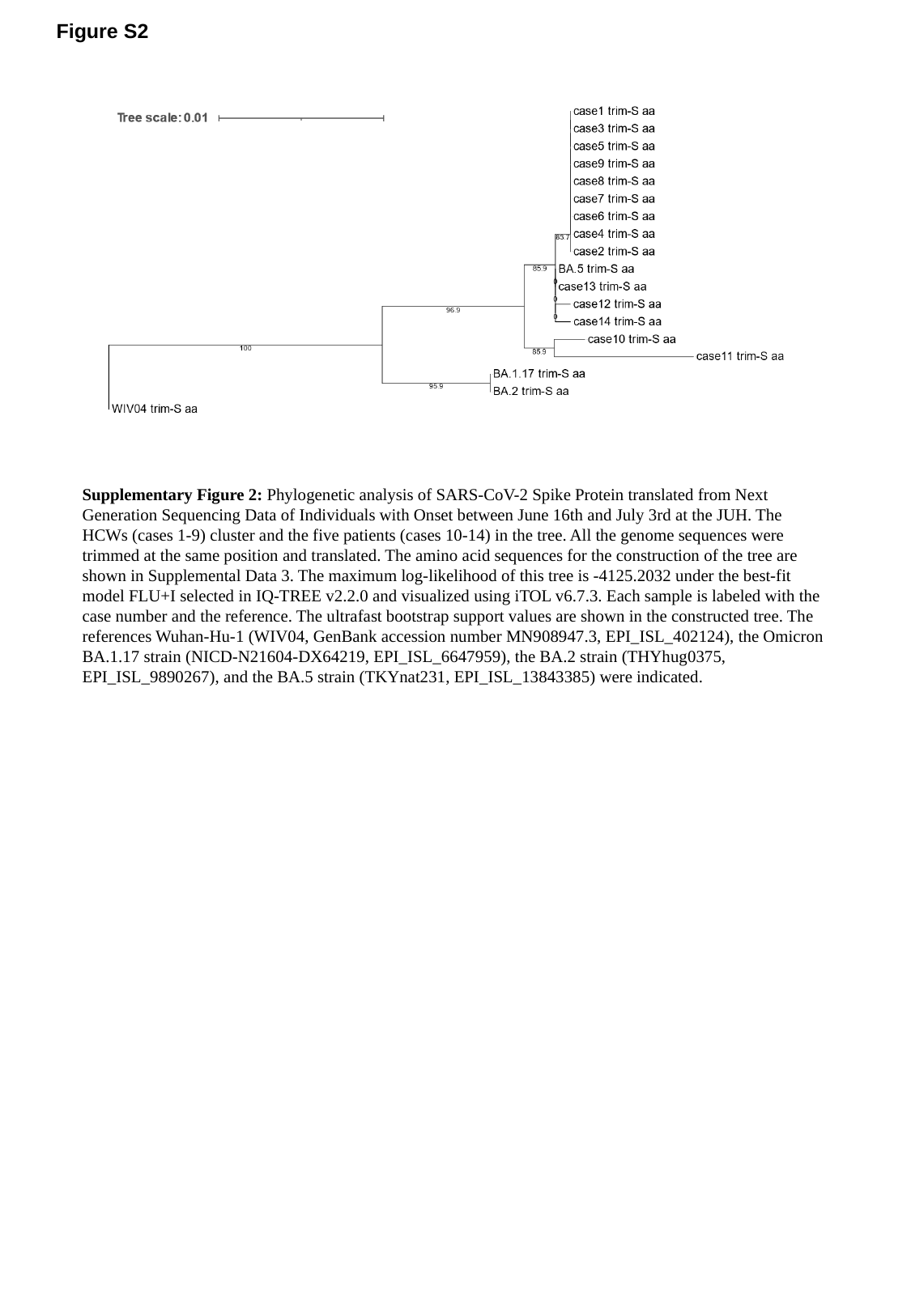

Figure S2
Supplementary Figure 2: Phylogenetic analysis of SARS-CoV-2 Spike Protein translated from Next Generation Sequencing Data of Individuals with Onset between June 16th and July 3rd at the JUH. The HCWs (cases 1-9) cluster and the five patients (cases 10-14) in the tree. All the genome sequences were trimmed at the same position and translated. The amino acid sequences for the construction of the tree are shown in Supplemental Data 3. The maximum log-likelihood of this tree is -4125.2032 under the best-fit model FLU+I selected in IQ-TREE v2.2.0 and visualized using iTOL v6.7.3. Each sample is labeled with the case number and the reference. The ultrafast bootstrap support values are shown in the constructed tree. The references Wuhan-Hu-1 (WIV04, GenBank accession number MN908947.3, EPI_ISL_402124), the Omicron BA.1.17 strain (NICD-N21604-DX64219, EPI_ISL_6647959), the BA.2 strain (THYhug0375, EPI_ISL_9890267), and the BA.5 strain (TKYnat231, EPI_ISL_13843385) were indicated.
